# Period multiplication cascade at the order-by-disorder transition in uniaxial random field XY magnets

**DOI:** 10.1038/s41467-020-18270-6

**Published:** 2020-09-16

**Authors:** S. Basak, K. A. Dahmen, E. W. Carlson

**Affiliations:** 1 Department of Physics and Astronomy, Purdue University, West Lafayette, IN 47907 USA; 2 Purdue Quantum Science and Engineering Institute, West Lafayette, IN 47907 USA; 3 Department of Physics, University of Illinois at Urbana-Champaign, Urbana, IL 61801 USA; 4 LPEM, ESPCI Paris, PSL Research University; CNRS; Sorbonne Université, 75005 Paris, France

**Keywords:** Phase transitions and critical phenomena, Nonlinear phenomena, Phase transitions and critical phenomena

## Abstract

Uniaxial random field disorder induces a spontaneous transverse magnetization in the XY model. Adding a rotating driving field, we find a critical point attached to the number of driving cycles needed to complete a limit cycle, the first discovery of this phenomenon in a magnetic system. Near the critical drive, time crystal behavior emerges, in which the period of the limit cycles becomes an integer *n* > 1 multiple of the driving period. The period *n* can be engineered via specific disorder patterns. Because *n* generically increases with system size, the resulting period multiplication cascade is reminiscent of that occurring in amorphous solids subject to oscillatory shear near the onset of plastic deformation, and of the period bifurcation cascade near the onset of chaos in nonlinear systems, suggesting it is part of a larger class of phenomena in transitions of dynamical systems. Applications include magnets, electron nematics, and quantum gases.

## Abstract (web summary)

In the XY model, the application of a disordered uniaxial magnetic field leads to a long range magnetic order. Here, using a numerical approach, the authors find that at a critical rotating driving field, the number of driving cycles needed to complete a limit cycle diverges.

## Introduction

The XY model, in which interacting spins are confined to rotate within a plane, has been a staple of statistical mechanics and condensed matter studies, having been applied to a broad range of physical systems, including planar magnets, superfluids, superconductors, two-dimensional (2D) melting, nematic liquid crystals, and electron nematics, among others^1–10^. In two dimensions, the XY model exhibits a Berezinskii–Kosterlitz–Thouless transition to a power-law ordered phase, yet with no long-range order^11,12^. As such, the addition of random fields to a 2D XY model is expected to result in even less order: Imry and Ma argued that a (*d* ≤ 4)-dimensional system with continuous order parameter (with O(*n*) symmetry with *n* ≥ 2) in the presence of random fields cannot have long-range order for any finite disorder strength^13^.

However, the addition of uniaxial random fields reduces the global symmetry of the Hamiltonian, and the Imry–Ma argument no longer applies^14^. In this case, the low temperature phase has long-range order via an order-by-disorder transition, in which XY spins align perpendicular to the random fields^14,15^. This is a special case of a more general class of order-by-disorder transition, where an *n*-dimensional spin system orders in a (*n*–*k*)-dimensional subspace due to orthogonal *k*-dimensional random fields^3,9,14,16^.

In this paper, we consider the possibility of a nonequilibrium transition. We use simulations to study the order-by-disorder transition in the presence of a rotating driving field at zero temperature. To our knowledge, this is the first time the uniaxial random field XY model has been studied in the presence of a rotating driving field. By analyzing the avalanche size distribution as a function of magnitude of applied driving field, we find evidence that the system undergoes a continuous nonequilibrium phase transition at a critical amplitude of the driving field. Once a limit cycle is established, we observe that the period of the hysteresis loops become *n*-fold near a critical applied field strength, where *n* is as large as 7 in our largest systems. We present evidence that the period of the subharmonic entrainment is rigid against perturbations in initial conditions and against perturbations of the drive field, indicating that a classical discrete time crystal emerges near criticality^17,18^. We present finite size scaling evidence that the period of these multi-period limit cycles will diverge in the thermodynamic limit. An experimental test of this would be the presence of non-repeatability in the response due to a rotating driving field near the transition.

As discussed further in the “Discussion” section, there are several experimental systems corresponding to the XY model into which uniaxial random field disorder can be incorporated, whereby these ideas can be tested experimentally. These include layers of Josephson junctions^1^, superfluid in a uniaxially stressed aerogel^2^, ultracold atoms in the presence of speckle radiation^3^, uniaxially stressed 2D Wigner crystals^4–7^, the half-integer quantum Hall effect^8^, and possibly the graphene quantum Hall ferromagnet^9,10^.

We consider the uniaxial random field XY model on a square lattice, in the presence of a driving applied field **H**, with constant magnitude *H* = ∣**H**∣:

1 $${\mathcal{H}}=-J\sum_{\langle i,j\rangle }\cos ({\theta }_{i}	-{\theta }_{j}) -{\sum }_{i}{h}_{i}\cos ({\theta }_{i})\\ 	-H{\sum }_{i}\cos ({\theta }_{i}-\phi ),$$

where ${{\bf{S}}}_{i}\equiv (\cos ({\theta }_{i}),\sin ({\theta }_{i}))$ is the XY spin on each site *i* and *J* is the nearest neighbor interaction strength. The second term arises from the interaction of a local random field along the *x*-axis and the XY spins. We choose the random field *h*_*i*_ at each site *i* from a Gaussian probability distribution of width *R*_*x*_, $P({h}_{i})=\exp [-{h}_{i}^{2}/(2{R}_{x}^{2})]/(\sqrt{2\pi {R}_{x}^{2}})$. The order parameter is the magnetization per site ${\bf{m}}=\frac{1}{N}\mathop{\sum }\nolimits_{i = 1}^{N}{{\bf{S}}}_{i}$, where *N* = *L* × *L* is the number of sites.

We study this system at zero temperature under the influence of a rotating applied driving field whose angle *ϕ* = *ωt* advances in time slowly, in the *ω* → 0 limit. The dynamics is quasi-static: after each small increment of the driving field angle, the energy of the system is minimized. (See the “Methods” section for details of the simulation method.) This type of dynamics^19^ presupposes that the system is connected to a heat bath, which prevents heating by the drive.

Symmetry considerations imply that the timescales associated with barriers to equilibration of this model diverge exponentially near criticality^20^, for the following reasons. In the presence of a uniform applied field *H*, the symmetry of the XY model is reduced to that of the Ising model. This means that the system can have a symmetry-breaking transition, in which a spontaneous magnetization forms perpendicular to the applied field *H*. Adding a uniaxial random field along any axis that is not parallel to *H* applies random fields to that Ising variable, placing the critical behavior in the universality class of the random field Ising model. It is well known that the timescales to equilibration diverge exponentially with proximity to criticality in the random field Ising model^20^. In fact, at the corresponding critical point, temperature fluctuations are irrelevant in the renormalization group sense, meaning they are not necessary in order to capture the essential critical behavior. During a single cycle of the rotating applied field we consider here, the symmetry of the system remains in the universality class of the random field Ising model, except for a set of measure zero (when *H* is parallel to the random fields). Therefore, as the applied field is rotated, the system is forced to traverse regions with enormous energy barriers most of the time. On long enough length scales, these energy barriers must be present. For a given rate of dissipation of the heat bath, the energy barriers can be made to diverge sufficiently to beat the rate of dissipation by moving closer to criticality. Thus we study our model at zero temperature, for the same reason that zero temperature results from the random field Ising model have been applied to many disparate physical systems, some even at room temperature^21^.

## Results

**Fig. 1:**
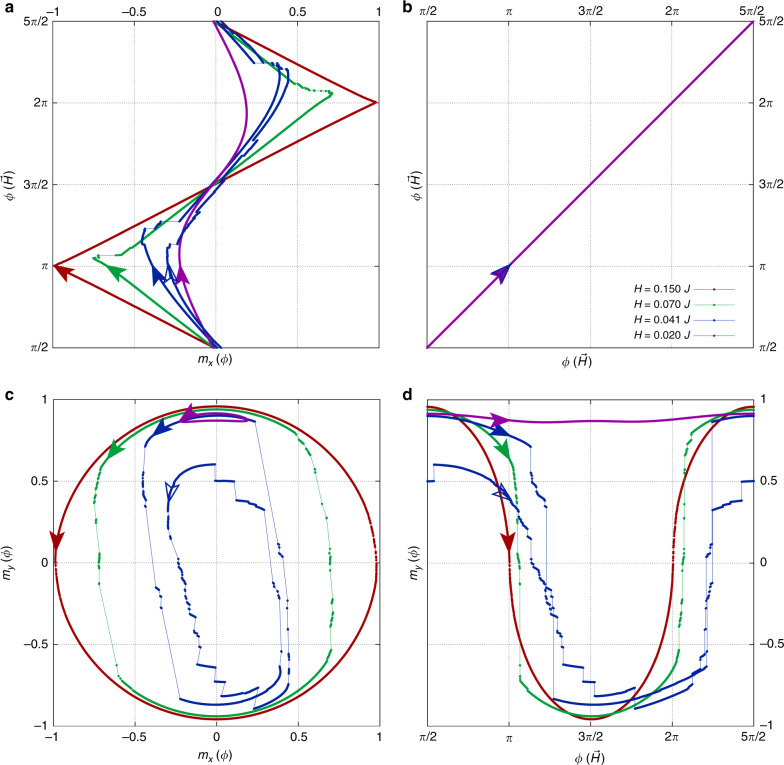
Steady-state response to rotating applied field at *T* = 0. A system of size *N* = 160 × 160 with *R*_*x*_ = 0.5*J* is started from an initial applied field in the *y*-direction. The initial spin configuration is aligned with the applied field, then relaxed according to Eq. (3) as described in the text, after which the applied field is rotated counterclockwise as denoted in **b**. **a**, **c**, **d** show the response once steady state is reached under the driving field. **a** shows the response of the magnetization in the *x* direction, while **d** shows the response of the magnetization in the *y* direction. **c** is a parametric plot of *m*_*y*_ vs. *m*_*x*_. In all panels, the arrows denote the state of the system when the driving field is at an angle *ϕ* = *π*, i.e., aligned along the *x* direction. For driving field strength *H* = 0.041*J*, the response of the system has double the period of the driving field. The open arrow on this trace denotes the state of the system at driving field angle *ϕ* = *π* during every other cycle of the driving field.

  Figure 1 shows the rich behavior of the limit cycles in rotating driving field, as a function of the magnitude of the driving field *H* at intermediate disorder strength *R*_*x*_ = 0.5*J*. Figure 1b shows the sense of the driving field, which is held at constant magnitude, but rotated counterclockwise, i.e., *ϕ* increases in time as *ϕ* = *ωt* in the *ω* → 0 limit, starting from *ϕ* = *π*/2. Figure 1a shows a plot of *m*_*x*_ vs. the angle *ϕ* of the applied field. Figure 1d shows a plot of *m*_*y*_ vs. the angle *ϕ* of the applied field. Figure 1c shows the combined parametric plot of magnetization *m*_*x*_ in the *x* direction, plotted against the magnetization *m*_*y*_ in the *y* direction. The sense of the parametric plot in Fig. 1c is counterclockwise. In each case, the system is started from a locally stable configuration in applied field ${\bf{H}}| | \hat{{\bf{y}}}$ at zero temperature, which has been relaxed from an initially saturated state aligned with the initial applied field. The transient response before the limit cycle is not shown in this figure. We return to the transient response later.

For moderate disorder strength *R*_*x*_ = 0.5*J*, we find that, at small amplitudes of the driving field, the spontaneous magnetization in the *y* direction remains robust. This is evident in the small hysteresis loops we find for *H* = 0.02*J* as shown by the purple trace in the parametric plot Fig. 1c. This indicates that the system continues to display spontaneous symmetry breaking in the *y* direction, retaining its Ising ferromagnetic character in the presence of weak rotating driving field.

As the magnitude of the applied field is increased, there is a change in behavior from ferromagnetic to paramagnetic response. This is evident in the large, almost circular hysteresis loop we find for larger *H* = 0.15*J*, as shown by the red trace in the parametric plot Fig. 1c. This change is consistent with either a crossover in behavior or a non-equilibrium phase transition at a critical magnitude of the driving field. Note that the rotating hysteresis loops at intermediate driving field strengths *H* = 0.041*J* and *H* = 0.07*J* have rich structure: Numerous avalanches are evident in these traces. As we discuss later, the avalanche structure provides further insight into the question of whether the change from ferromagnetic to paramagnetic response is a crossover or a phase transition. Perhaps the most intriguing feature of the intermediate driving field regime is that, in the blue trace (*H* = 0.041*J*), the limit cycle has double the period of the driving field. We find that limit cycles often become multiperiodic at intermediate field strength, for large enough system size.

In this section, we focus on the characteristics of the avalanches that occur near the transition from Ising ferromagnetic to paramagnetic response. We find a rich avalanche structure at intermediate field strengths, as can be seen in the blue and green traces in Fig. 1 (*H* = 0.041*J* and *H* = 0.07*J*, respectively). Notice that, while the avalanches are apparent in both *m*_*x*_ and *m*_*y*_, they are most prominent in *m*_*y*_, which serves as the order parameter in this system. When magnetization is cast as an extensive quantity, ${\bf{M}}=\mathop{\sum }\nolimits_{i = 1}^{N}{{\bf{S}}}_{i}=N{\bf{m}}$, then in the thermodynamic limit, avalanches *δ***M** of diverging size accompany a second-order phase transition.

**Fig. 2:**
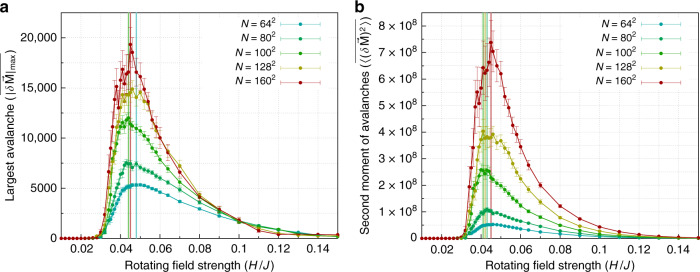
Avalanche statistics for disorder strength *R*_*x*_ = 0.5*J* from zero temperature simulations. The response of the magnetization to rotating driving field often proceeds via avalanches, in which there is a discontinuous jump in the magnetization *δ***M** in response to a small change *δϕ* of the driving field angle. **a** Statistics of largest avalanches. In **a**, we plot the size of the largest avalanche $| \delta {\bf{M}}{| }_{\max }$ per limit cycle at each rotating field strength, disorder averaged, for a range of system sizes. **b** Statistics of second moment of avalanches. **b** shows the disorder average of the second moment *δ***M** of the avalanche size distribution where the error bars are the standard deviation over the disorder average as described in the text. The brackets $<\ >$ denote an average over the limit cycle, and the overbar denotes a disorder average. By both of these measures, the size of the avalanches grows with system size implying divergent fluctuations at a critical field strength in the thermodynamic limit. The vertical bars in both panels mark the peak value from a running 3-point average. Within the resolution of the plot in **a**, these values are coincident for sizes *N* = 80 × 80 and *N* = 100 × 100, and for sizes *N* = 128 × 128 and *N* = 160 × 160. In **b**, the peak values are coincident for sizes *N* = 64 × 64 and *N* = 160 × 160.

  Figure 2a plots the size of the largest avalanche $| \delta {\bf{M}}{| }_{\max }$ at each rotating field strength, for a range of system sizes *N* = *L* × *L*. Results are averaged over several disorder configurations of the random field at disorder strength *R*_*x*_ = 0.5*J*, ranging from 75 disorder configurations for system size *N* = 64^2^ to 30 disorder configurations for system size *N* = 160^2^. (See “Methods.”) Notice that fluctuations as measured by the largest avalanche diverge with increasing system size at a critical driving field strength, *H*_c_(*R*_*x*_ = 0.5*J*). We estimate the value of *H*_*c*_ at *R*_*x*_ = 0.5*J* as follows: For each system size, the peak value based on a 3-point average is indicated by the vertical bar. The corresponding peak value of the applied field strength, averaged over all system sizes, is *H*_c_ = (0.0452 ± 0.0015)*J*.

In Fig. 2b, we plot the second moment of all avalanches in each limit cycle, $<{(\delta {\bf{M}})}^{2}>$ at each rotating driving field strength, for a range of system sizes. Results are disorder averaged, using the same number of disorder configurations as in Fig. 2a. Notice that this alternate measure of fluctuations based on the second moment of the avalanche size distribution is also consistent with the system undergoing a second order, nonequilibrium phase transition at a critical driving field strength, *H*_c_. In this case, we find that *H*_c_(*R*_*x*_ = 0.5*J*) = (0.0432 ± 0.0016)*J*, in agreement with the value of the critical field strength we find from Fig. 2a.

**Fig. 3:**
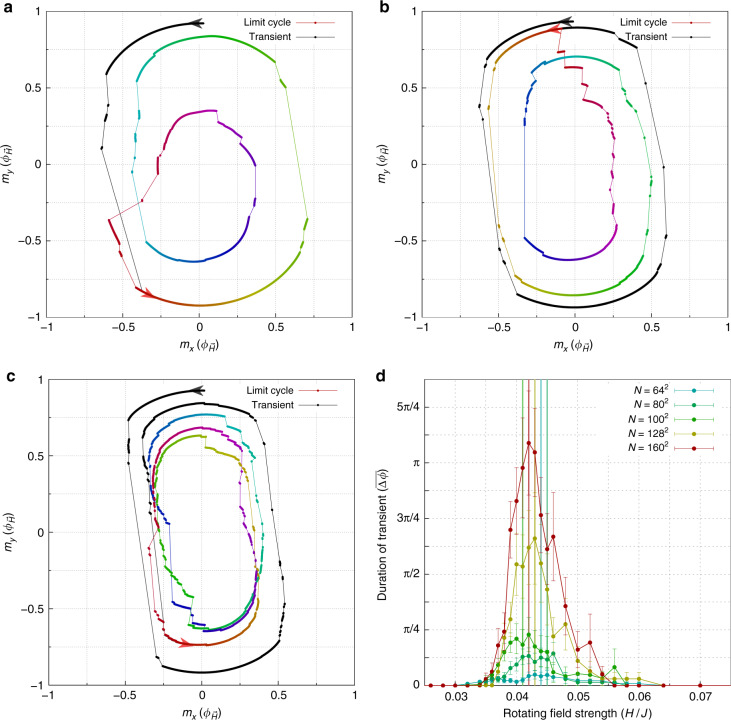
Transient response and multiperiod limit cycles at *T* = 0 near the transition field strength for disorder strength *R*_*x*_ = 0.5*J*. **a**–**c** show the initial transient response (black curves), followed by multiperiodic limit cycles (rainbow curves). **a** *H* = 0.058*J*; *N* = 64 × 64. Transient response and multiperiodic limit cycle for one disorder configuration at *N* = 64^2^. Here the transient response lasts roughly half a cycle before a period-2 limit cycle is established. **b** *H* = 0.048*J*; *N* = 100 × 100. Transient response and multiperiodic limit cycle for one disorder configuration at a larger system size *N* = 100^2^. Here the transient response lasts roughly one cycle before a period-2 limit cycle appears. **c** *H* = 0.046J; *N* = 160 × 160. Transient response and multiperiodic limit cycle for one disorder configuration at an even larger system size *N* = 160^2^. Here the transient response lasts almost 1.5 cycles before a period-3 limit cycle is established. **d** Statistics of transient response. The disorder-averaged duration of the transient response, as a function of *H*. The error bars are the standard deviation over the disorder average as described in the text. The mean of the transient distribution function for each system size is marked by a vertical line of the corresponding color.

Figure 3a–c shows how the magnetization responds to a rotating driving field in the vicinity of the phase transition. There is a transient response before the system settles into a limit cycle. A limit cycle is the steadily repeating response in the magnetization due to a rotating driving field. While we find that most limit cycles have the same period as the driving field, we find that near the transition regime, limit cycles often have a longer period. We first discuss the behavior of the transient response, before turning our attention to the behavior of the multiperiodic limit cycles.

The transient response in Fig. 3a–c is marked in black. In Fig. 3d, we plot the duration of the transient response, as a function of *H*, for various system sizes. The results shown have been averaged over several disorder configurations. (See “Methods” for details.). At high and low strengths of the driving field, the transient response becomes so negligible as to be smaller than the symbol size on this graph. However, at intermediate driving field strength, the transient response grows with increasing system size. The fact that the transient response grows with increasing system size is further corroboration that the system is undergoing a second-order phase transition. In Fig. 3, the mean of each transient distribution function is denoted by a vertical line, color coded to the system size. The average of the mean value of *H* from these vertical lines is ${\langle {H}_{{\rm{tr}}}\rangle }_{N}=(0.0430\pm 0.0014)J$, consistent with our previous estimates of *H*_c_(*R*_*x*_ = 0.5*J*).

**Fig. 4:**
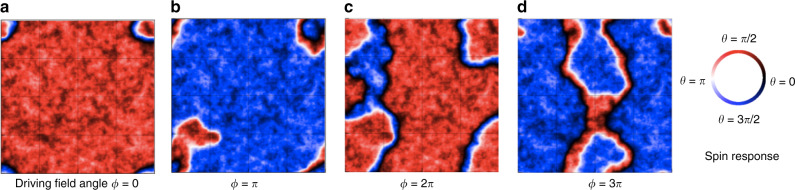
Example of spin configurations during a period-2 limit cycle at zero temperature. Spin configurations **a** and **c** are for the same angle *ϕ* of the driving field, but the spin configuration is different the second time through the driving cycle. Likewise, spin configurations **b** and **d** are for the same angle *ϕ* of the driving field, but the spin configuration is different the second time through the driving cycle. For this particular disorder configuration and system size, the spin configurations repeat every 2 periods of the driving cycle. Here the driving field strength is *H* = 0.04*J*, and the system size is 160 × 160. See Fig. 1 of Supplementary Information and videos^22^ of simulation results for further examples.

We now turn our attention to the behavior of the limit cycles at intermediate driving field strength. One of the most fascinating features of the limit cycles in this regime is that some of them have a longer period than that of the driving field. Figure 3 shows some representative cases of this behavior. Figure 4 visualizes how the spin configurations respond to the driving field during one of the period-2 limit cycles. Domain walls have dramatically different configurations during the second cycle as opposed to the first cycle of the driving field, suggesting a prominent role for domain wall pinning and domain wall creep. More examples of such behavior can be found in Fig. 1 of the Supplementary Information and in our videos^22^ of the simulation results.

**Fig. 5:**
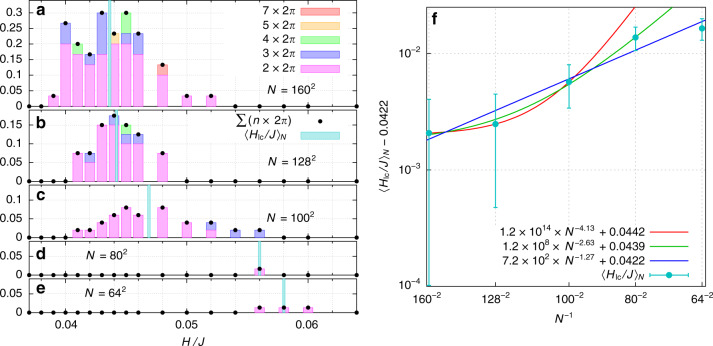
Multiperiod limit cycles at zero temperature. **a**–**e** show what fraction of limit cycles that exhibit multiperiodicity as a function of driving field strength *H* at *R*_*x*_ = 0.5*J*. The smallest system size we simulated, *N* = 64 × 64, is shown on the bottom left in **e**. System size increases from bottom to top in the left panels, up to system size *N* = 160 × 160. In the bar graphs, period-2 limit cycles are shaded pink; period-3 limit cycles are purple; period-4 limit cycles are green; the period-5 limit cycle is orange; and the period-7 limit cycle is red. We did not observe any period-6 limit cycles. Black dots represent the net contribution from all multiperiod limit cycles at each field. In each bar graph, the vertical blue line is the mean of the distribution function, $<{H}_{{\rm{lc}}}>$ in units of *J*. **f** From the results of **a**–**e**, we plot ${\langle {H}_{{\rm{lc}}}\rangle }_{N}$ vs. the inverse of system size *N* on a log–log scale. In **f**, the error bars are standard deviations over the histograms in **a**–**c**, **e**, respectively. For the fourth point (arising from **d**), we estimate the error to be the average of that in the third and fifth points. A power law fit of ${\langle {H}_{{\rm{lc}}}\rangle }_{N}$ for the three largest system sizes is given by the red curve; the fit for the four largest system sizes is given by the green curve; and the fit for all calculated system sizes is given by the dark blue curve. The *y*-intercept is consistent among all of these fits, yielding an average value of ${\langle {H}_{{\rm{lc}}}\rangle }_{N\to \infty }=(0.0434\pm 0.0020)J$.

In order to explore this behavior quantitatively, we studied several disorder configurations near the transition, as a function of system size. Figure 5 shows a histogram of the likelihood of multiperiod limit cycles. For a given magnitude of the driving field *H* and a given system size *N*, we plot the number of disorder configurations whose limit cycle has a period greater than that of the driving field divided by the number of all disorder configurations studied at that *H* and *N*. Starting from the bottom panel on the left-hand side of Fig. 5e, the system size increases as one moves to the next panel up the page, up to Fig. 5a that shows the largest system we studied, *N* = 160 × 160. Different color bars indicate the period of the multiperiod behavior: pink indicates period doubling; blue shows period tripling; period-4 limit cycles are denoted in green; yellow is for period-5, and orange is for period-7. We did not observe any period-6 limit cycles, although presumably these would appear at certain disorder configurations as well.

The vertical blue bars mark the mean of the distributions in Fig. 5a–e, ${\langle {H}_{{\rm{lc}}}\rangle }_{N}$. In Fig. 5f, we plot ${\langle {H}_{{\rm{lc}}}\rangle }_{N}$ vs. 1/*N* on a log–log plot, in order to determine the limiting value ${\langle {H}_{{\rm{lc}}}\rangle }_{N\to \infty }$. Fits of the finite size scaling in Fig. 5f for all system sizes, the four largest system sizes, and the three largest system sizes yield a consistent value for ${\langle {H}_{{\rm{lc}}}\rangle }_{N\to \infty }$ within error bars. The average of these three methods yields ${\langle {H}_{{\rm{lc}}}\rangle }_{N\to \infty }=(0.0434\pm 0.0020)J$.

**Fig. 6:**
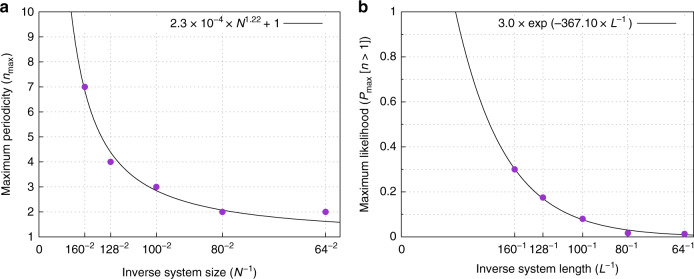
Trends of the zero temperature multiperiodic behavior of the limit cycles with increasing system size. **a** Maximum period of limit cycles. In **a**, we plot the maximum period of the limit cycles observed in Fig 5a–e, as a function of 1/*N* (purple circles). The black line is a fit to the simulation results. The trend is toward divergence of the period of limit cycles in the thermodynamic limit. **b** Maximum likelihood of multiperiod limit cycles. In **b**, we plot the maximum likelihood of multiperiodic limit cycles, obtained from the peak heights of the left-hand panels in Fig. 5 (purple circles). The black line is a fit to the simulation results. The trend is toward saturation of the likelihood of multiperiod behavior in the thermodynamic limit.

We find that, at small system size, multiperiod behavior is rare. However, as the system size is increased, and the disorder configurations can become correspondingly more rich, the likelihood of multiperiod behavior increases. In Fig. 6a, we plot the maximum observed period of a limit cycle vs. 1/*N*. The maximum period increases with increasing system size, in a manner consistent with diverging period in the thermodynamic limit.

Notice also that the distribution in Fig. 5a–e grows in height with increasing system size. For *N* = 160 ×  160, we find that 20–30% of disorder configurations in the range *H* = (0.04 − 0.046)*J* display multiperiodic behavior. To quantify these effects, we plot the maximum height of the distributions in Fig. 5a–e in Fig. 6b. This measure also shows sharp increase with increasing system size. The fact that both the likelihood of multiperiod behavior and the period of limit cycles steadily increase with increasing system size points toward a thermodynamic limit in which the period of limit cycles goes to infinity. If the period of a system diverges in the thermodynamic limit, then the system has effectively entered a regime of non-repeatability. We discuss further implications of this finding in the next section.

## Discussion

**Fig. 7:**
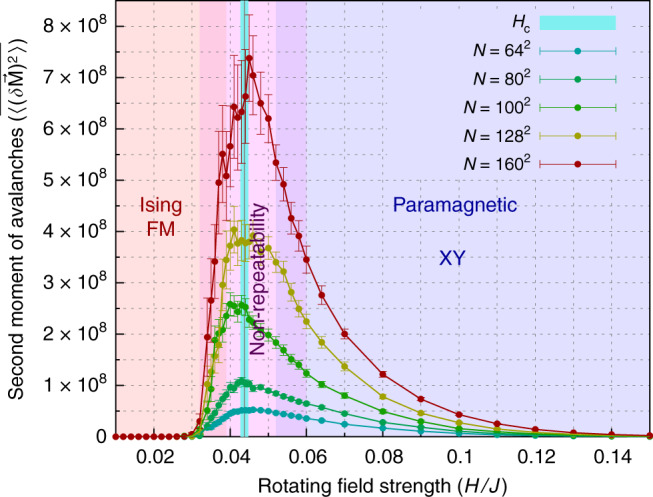
This figure shows the zero temperature phase diagram as a function of the strength of the rotating field in a uniaxial random field. The region where the number of multiperiodic loops and the maximum periodicity increases with system size is labeled as the expected region of non-repeatability for infinitely large systems. This region coincides with the region where the largest avalanche occurs is this system where 〈*H*_c_〉 = (0.0437 ± 0.0009)*J*, which is marked by the vertical blue line. The line plots shows that the disorder average of the second moment *δ***M** of the avalanche size distribution where the error bars are the standard deviation over the disorder average as described in the text. The brackets $<\,\,>$ denote an average over the limit cycle, and the overbar denotes a disorder average.

Using four different methods to quantify the fluctuations in the system (see Table 1), we find evidence for a second-order nonequilibrium phase transition from spontaneous Ising ferromagnetism at low driving field strength to XY paramagnetism at high driving field strength. The critical field strength at which this transition occurs is consistent across all the methods we employed, yielding an average value of *H*_c_ =  (0.0437 ± 0.0009)*J*, as denoted in the phase diagram in Fig. 7.

**Table 1:** Critical field strength.

Method	Value of *H*_c_/*J*
Largest avalanche of limit cycle	0.0452 ± 0.0015
Second moment of avalanches in limit cycle	0.0432 ± 0.0016
Duration of transient response	0.043 ± 0.0014
Finite size scaling of multiperiodic behavior	0.0434 ± 0.0020
Overall average of the above methods	0.0437 ± 0.0009

We furthermore find that, far from being irrelevant, disorder plays a prominent role at the transition. Because the disordered energy landscape makes the system highly susceptible to spatial fluctuations near the transition, there is both a longer transient response and a longer period of limit cycles near *H*_c_. Remarkably, both the likelihood of multiperiod behavior and the period of the limit cycles increases with no sign of saturation as system size is increased. The trend we find is toward a thermodynamic limit in which limit cycles never repeat. A large enough physical system at this critical point should therefore display a regime of non-repeatability. As shown in Fig. 7, the regime of non-repeatability in the thermodynamic limit coincides with the nonequilibrium phase transition. The dependence of this simple model upon history implies that experiments on XY systems in uniaxial random field are particularly sensitive to disorder. Conflicting experimental results could arise if hysteresis protocols are not closely monitored.

Similar behavior is predicted to occur in models of amorphous solids under periodic shear stress^19,23,24^. In these systems, simulations revealed that under periodic shear the response of the system becomes multiperiodic, in a way that is consistent with chaotic behavior at a critical shear amplitude. More work would be needed to determine whether the multiperiodic cascade observed here is indicative of chaotic behavior in the thermodynamic limit. Similar multiperiod cascades signal the onset of chaos in nonlinear systems, suggesting that the multiperiod cascades observed here and in periodically driven models of amorphous solids are characteristic of a larger class of transitions in dynamical systems.

While the discussion above points toward non-repeatability in the thermodynamic limit, there is a way to take the thermodynamic limit on this model such that *n* remains finite. A finite system of size *L* × *L* with a particular disorder pattern at the critical point has a finite period *n* with respect to the driving period. Now tile space by making *k* copies of this system (including the particular disorder pattern), and let *k* → *∞*. With this method of taking the thermodynamic limit, the period *n* remains finite, even for increasing system size. We have verified that when a disorder pattern is tiled into a 2 × 2 superlattice of the original disorder pattern (i.e., *k* = 4) then the spin response in the limit cycle is also a superlattice of the original spin configuration, and *n* is unchanged from the case *k* = 1.

By this second method of taking the thermodynamic limit, in the vicinity of the nonequilibrium transition, the system should display the characteristics of a classical^17,18^ discrete time crystal^25–27^, in which the discrete time translation symmetry imposed by the periodic drive is spontaneously broken in a way that leads to rigid subharmonic entrainment. While some authors are willing to apply the label time crystal to an open system, where energy from the drive moves through the system into a heat bath (as in the present case)^17,26,28^, others prefer a more restrictive use of the term time crystal, reserving it for closed, conservative systems^29^. We are using the term time crystal in the former, broader sense.

We find that the period of the response remains stable against perturbations in the initial conditions and stable against low temperature fluctuations (see Section B in Supplementary Information), indicating that the spontaneous breaking of the discrete time symmetry is rigid. Yao et al.^17^ find that the critical endpoint between a classical discrete time crystal and the disordered phase of a dissipative, coupled chain of classical nonlinear pendula terminates in a critical point that is not in an Ising universality class. Because the nonequilibrium transition we find here is in an Ising universality class, this indicates that there is more than one classical discrete time crystal universality class. The results here further underscore the fact that long-range interactions are not a necessary ingredient to stabilize a time crystal^30^.

While our results point to rigidity of *n* with respect to very low temperature fluctuations, more analysis would be needed to establish whether the *n* is truly long-range ordered in time. However, what we observe is a promising avenue toward time crystal behavior in a new system, as can be seen in Fig. 4. The figure shows a period-2 limit cycle. Two types of domain walls are evident in the figure: single domain walls that are either white or black, and double domain walls that are white and black. Comparing Fig. 4a and Fig. 4c, it is evident that, on the second time through the driving cycle, the domain walls are in a very different configuration as compared to the first time through the drive cycle. The same is true comparing Fig. 4b and Fig. 4d. Furthermore, the single (white) domain wall in the lower left of Fig. 4b has no counterpart in Fig. 4d and is topologically distinct from it. These are all indications that (i) the domain walls are pinned by the random fields, and therefore that (ii) spin configurations in the second cycle likely have high energy barriers to spin configurations at the same phase of the drive during the first cycle. In random field models, the timescales to equilibration grow exponentially near criticality^20^. This combination of topological differences, domain wall pinning, and high barriers to equilibration is the physical origin of the stability of the period of these multiperiod cycles against low temperature fluctuations and likely leads to true time crystalline rigidity of *n* if care is taken in how the thermodynamic limit is approached and how dissipation is handled.

The work in this paper was done at uniaxial random field strength *R*_*x*_ = 0.5*J*, with zero random field strength in the *y*-direction. Further work is needed to obtain the full phase diagram as a function of random field strengths *R*_*x*_ and *R*_*y*_.

The uniaxial random field XY model has been applied to many systems, including layers of Josephson junctions,^1^ superfluid in a uniaxially stressed aerogel,^2^ ultracold atoms in the presence of speckle radiation,^3^ uniaxially stressed 2D Wigner crystals,^4–7^ and the half-integer quantum Hall effect^8^. Uniaxial random field-induced order has also been discussed in connection with the graphene quantum Hall ferromagnet^9,10^. We discuss below a few of these systems in which there is also a clear way to drive the system with a rotating field.

An electron nematic occurs when the electronic degrees of freedom spontaneously break the rotational symmetry of the host crystal. Electron nematics have been observed or proposed in several material systems, including transition metal oxides like cuprate superconductors, manganites, nickelates, and cobaltites and valley symmetry breaking systems like single and bilayer graphene, elemental bismuth, and AlGaAs 2D electron gases, as well as strontium ruthenates and iron pnictides^31,32^. For electron nematics with XY symmetry^33^, there is a factor of two between the physical angle of the nematic in the plane and the natural angles in an XY model. This is because a nematic is symmetric under 180° rotation, whereas the XY spins change sign under the same operation. The uniaxial random fields we discuss in this paper can arise in these systems if random orienting fields are strong only along the major crystalline axes. Note that, in this case, the order-by-disorder transition would induce the electron nematic to orient along a direction that is diagonal to the major crystalline axes.

Several external perturbations can be used as a driving field on an electron nematic, including magnetic field, electric field, high currents, and uniaxial stress^34,35^. Note that similar symmetry considerations apply to the driving field in these systems. For example, a rotating applied magnetic field ${\bf{B}}=[{B}_{x},{B}_{y}]=B[\left.\cos (\omega t)\right),\sin (\omega t)]$ can be used to exert the rotating driving field of Eq. (1) for the case of a nematic, with the caveat that rotating the applied field by 90° changes the sign of the driving field:

2 $${\bf{H}}=[{H}_{x},{H}_{y}]=H[\cos (2\omega t),\sin (2\omega t)]\,.$$

Random field-induced order has been proposed to happen in coupled Bose–Einstein condensate systems^3^. Theoretical and numerical results on two-component Bose gases predict that, by using a Raman field to couple two internal states, uniaxial random field disorder can be produced. The uniaxial nature is achieved by a Raman coupling with constant phase, while the randomness is achieved through random strength of the Raman field^36,37^. Similarly, a rotating driving field can be applied by a Raman coupling with uniform strength, but rotating phase.

While the mapping of a magnetic system with XY symmetry to Eq. (1) is clear, the realization of a uniaxial random field in these systems is less clear. It may be possible to design a system in which epitaxial strain from a substrate exerts random uniaxial fields on a 2D XY ferromagnet through magnetoelastic coupling.

In conclusion, we have shown that the order-by-disorder transition of the 2D XY model in the presence of a uniaxial random field persists up to a critical strength of the rotating driving field. Near the critical driving field strength, the response of the system has a period that is an integer multiple *n* > 1 of the driving field period. The trend with increasing system size is toward increasing period *n*, suggesting the onset of what is effectively non-repeatability as *n* → *large* in the thermodynamic limit. Similar multiperiod cascades signal the onset of chaos in nonlinear systems and signal the onset of irreversibility in periodically driven models of plastic deformation, suggesting that multiperiod cascades are characteristic of a larger class of transitions in dynamical systems. Our results further indicate that the period *n* can be engineered to remain finite if the thermodynamic limit is taken by tiling a particular disorder pattern into a superlattice. In this case, behavior reminiscent of classical discrete time crystals emerges near criticality.

## Methods

### Hysteresis protocol

The magnetization *m*_*y*_ in the *y*-direction at intermediate disorder strength *R*_*x*_ = 0.5*J* remains ordered even in the presence of weak applied transverse field *H*_*x*_. (See Section D in Supplementary Information.). Therefore, to begin the hysteresis studies, we first initialize the system in a *y*-magnetized state, by starting from the fully saturated *y* magnetization, with the driving field aligned along *y*, **H**∣∣*y*, then allow the system to relax^38^ at that applied field. We take the angle *ϕ* of the applied field to be *ϕ* = Arctan(*H*_*y*_/*H*_*x*_), so the initial direction of the applied field is *ϕ* = *π*/2. After rotating the applied field by an amount *δϕ*(**H**), the spin configuration is updated successively so as to minimize the energy, in the *ω* → 0 limit. After a transient response, the response of the system then settles into a limit cycle. Each time the applied field direction is updated, the energy is minimized on each site by aligning the spin on each site with its effective field, ${{\bf{h}}}_{i}^{{\rm{eff}}}$. Hence, the following update strategy is repeated until the spin configuration converges to the nearest energy minimum:

3 $${{\bf{h}}}_{i}^{{\rm{eff}}}(t)=	 \; J{\sum }_{j\in \langle i,j\rangle }{{\bf{S}}}_{j}(t)+{{\bf{h}}}_{i}+{\bf{H}},\\ {{\bf{S}}}_{i}(t+1)=	 \; \frac{{{\bf{h}}}_{i}^{{\rm{eff}}}(t)}{| {{\bf{h}}}_{i}^{{\rm{eff}}}(t)| }.$$

This update mechanism is similar to Eq. (2) of ref. ^39^; however, the effective on-site field in our case includes only the instantaneous influence of nearest neighbors, whereas ref. ^39^ is working in a mean-field limit. The update algorithm we employ is described in more detail below, in the next section.

We continue to allow spins to relax under the influence of Eq. (3) until a limit cycle is reached, defined by {**S**_*i*_}(*ϕ* + 2*πn*) = {**S**_*i*_}(*ϕ*). We use the following parameters in our simulations: *δm*_cutoff_ = 10^−4^, $\delta {\phi }_{\max }=2\pi \times 1{0}^{-4}$, $\delta {\phi }_{{\rm{min}}}={2}^{-14}\times \delta {\phi }_{\max }$. Hence the avalanches (*δm*) are only well defined within the precision of the driving field angle, $\delta {\phi }_{\min }=2\pi \times 6.1\times 1{0}^{-9}$.

### Spin relaxation method

The rotation of the driving field and subsequent relaxation of the spin configuration is performed as follows. Starting from an initial spin state {**S**_*i*_}(*ϕ*) for a given applied field direction *ϕ* = Arctan(*H*_*y*_/*H*_*x*_) and with *δϕ* initially set to *δϕ* = *δϕ*_max_:

- Update *ϕ* → *ϕ* + *δϕ*.
- Use Eq. (3) to relax the spin configuration.
- If *δm* > *δm*_cutoff_, then:
  - If *δϕ* = *δϕ*_min_, accept the new spin configuration and the new *ϕ* and proceed to Step 1.
  - Else reject the changes. Set *δϕ* → *δϕ*/2 and proceed to Step 1.
- Else accept the new spin configuration and the new *ϕ* and:
  - If $\delta \phi =\delta {\phi }_{\max }$ or $\delta m\ge \frac{\delta {m}_{{\rm{cutoff}}}}{2}$, proceed to Step 1.
  - Else, set *δϕ* → 2 × *δϕ* and proceed to Step 1.

### Disorder averages

  Table 2 reports the number of disorder configurations used in Figs. 2, 3, and 5.

**Table 2:** Number of disorder configurations used in Figs. 2a, b, 3d, and 5a–e.

Size (*N* = *L* × *L*)	Configurations
64 × 64	75
80 × 80	60
100 × 100	50
128 × 128	40
160 × 160	30

## Supplementary information

Supplementary Information

Supplementary Movie 1

Description of Additional Supplementary Files

## Data Availability

The numerical results that support the findings of this study are available from the corresponding author upon reasonable request.

## Code availability

The custom code used to generate the simulation results in the current study is available from the corresponding author upon reasonable request.
