# Supplementary material for: Period multiplication cascade at the order-by-disorder transition in uniaxial random field XY magnets
Source: Nat Commun. 2020 Sep 16;11:4665. doi: 10.1038/s41467-020-18270-6 (PMC7495492; doi:10.1038/s41467-020-18270-6)
Supplement: Supplementary file 1 — Supplementary Information [file 41467_2020_18270_MOESM1_ESM.pdf]

Period Multiplication Cascade at the Order-by-Disorder Transition in Uniaxial Random Field XY Magnets

Supplementary Information

Basak et al.

Supplementary Information for "Period Multiplication Cascade at the Order-by-Disorder Transition in Uniaxial Random Field XY Magnets"

S. Basak,[1,2] K. A. Dahmen,[3] and E. W. Carlson[1,2,*]

[1]*Department of Physics and Astronomy, Purdue University, West Lafayette, IN 47907, USA*
[2]*Purdue Quantum Science and Engineering Institute, West Lafayette, IN 47907, USA*
[3]*Department of Physics, University of Illinois at Urbana-Champaign, Urbana, IL 61801, USA*

(Dated: July 31, 2020)

A. Spin configurations under driving rotating field

In our zero temperature simulations, energy is minimized for each site based on the local field and the configuration of the nearest neighbor interactions. We use two types of driving protocol: one is changing the driving field angle (ϕ) at a constant rate; the other one is a variable rate where the rate is slowed down if the change in response magnetization is large and sped up if the response is small. Both these protocol gives us the same periodicity of the limit cycle. For example, if the constant rate is too large it can merge two avalances into one but the overall magnetization remains the same.

We also observe that the system falls into the same limit cycle however we initialize the spins. Due to the emergent Ising symmetry in the system and the above observation the limit cycles will be the same irrespective of the sense of rotating driving field. This is because the spin configurations can be mapped by a symmetry transformation from the response limit cycle of a clockwise rotating field to the response limit cycle of counterclockwise rotating field. Only the transient response depends on the initial spin configuration.

Figure 1 shows the various spin configurations the system goes through before and during a limit cycle with periodicity 4π. The rich structure of the domain walls are stable due to the random field distribution. All the plots in Fig. 1 are unique and Figs. 1(a-i) does not repeat but Figs. 1(j-cc) are part of the limit cycle which repeats indefinitely. See [1] for better visualizations of the evolving spin configurations in limit cycles with $n > 1$ periods.

B. Response with fluctuations in temperature

In order for a physical system to remain at very low temperature under the influence of a driving field, it is necessary for it to be connected to a low temperature heat bath which carries away the heat generated by the driving field in an efficient manner. This implies that the physical system will experience temperature fluctuations which arise from the heat bath. We model these temperature fluctuations with a Monte Carlo sweep in between spin relaxation. As long as fluctuations which arise due to temperature are low compared to the relaxation times of the system, this is a reasonable model for very low temperatures. We find (Fig. 2) that the multiperiod limit cycles are still rigid against the low temperature fluctuations which are simulated by the following protocol:

1. Initialize spins to fully ordered in the $+y$ direction. Set $\phi = \pi/2$.
2. After updating ϕ:
   (a) Update spins using the spin relaxation method described in the main text (Sec-IV).
   (b) One Monte-Carlo sweep at temperature ($T = 0.1J$) over the whole lattice using Glauber dynamics with checkerboard updates (This comprises one checkerboard update over all the black sites followed by all the white sites).
   (c) Update spins using the spin relaxation method again.
3. Update $\phi \to \phi + \delta\phi (= 0.0001 * 2\pi)$. Go to step-2.

This implies that the classical discrete Time Crystal(CDTC) we found is robust against small temperature fluctuations. Our CDTC is also interesting due to the fact that it has only short range interactions. This system is glassy, with large energy barriers. Therefore, given a certain rate of dissipation via the heat bath, the system can always be driven slowly enough that the rate of heating by the drive is well below the rate of cooling by the heat bath.[2, 3]

C. Gaussian Random Fields

For uniaxial random field disorder in the x direction, a local random field $h_{x,i}$ is chosen at each site i from a Gaussian distribution:

$$P(h_{x,i}) = \frac{1}{\sqrt{2\pi R_x^2}} \exp(-\frac{h_{x,i}^2}{2R_x^2}) \quad (1)$$

Because this is an unbounded probability distribution, the question arises as to whether we have accurately captured the behavior of the system in the presence of "rare events". To quantify the likelihood of a rare event, we ask the question: how large of a system size $N = L \times L$ do

* ewcarlson@purdue.edu

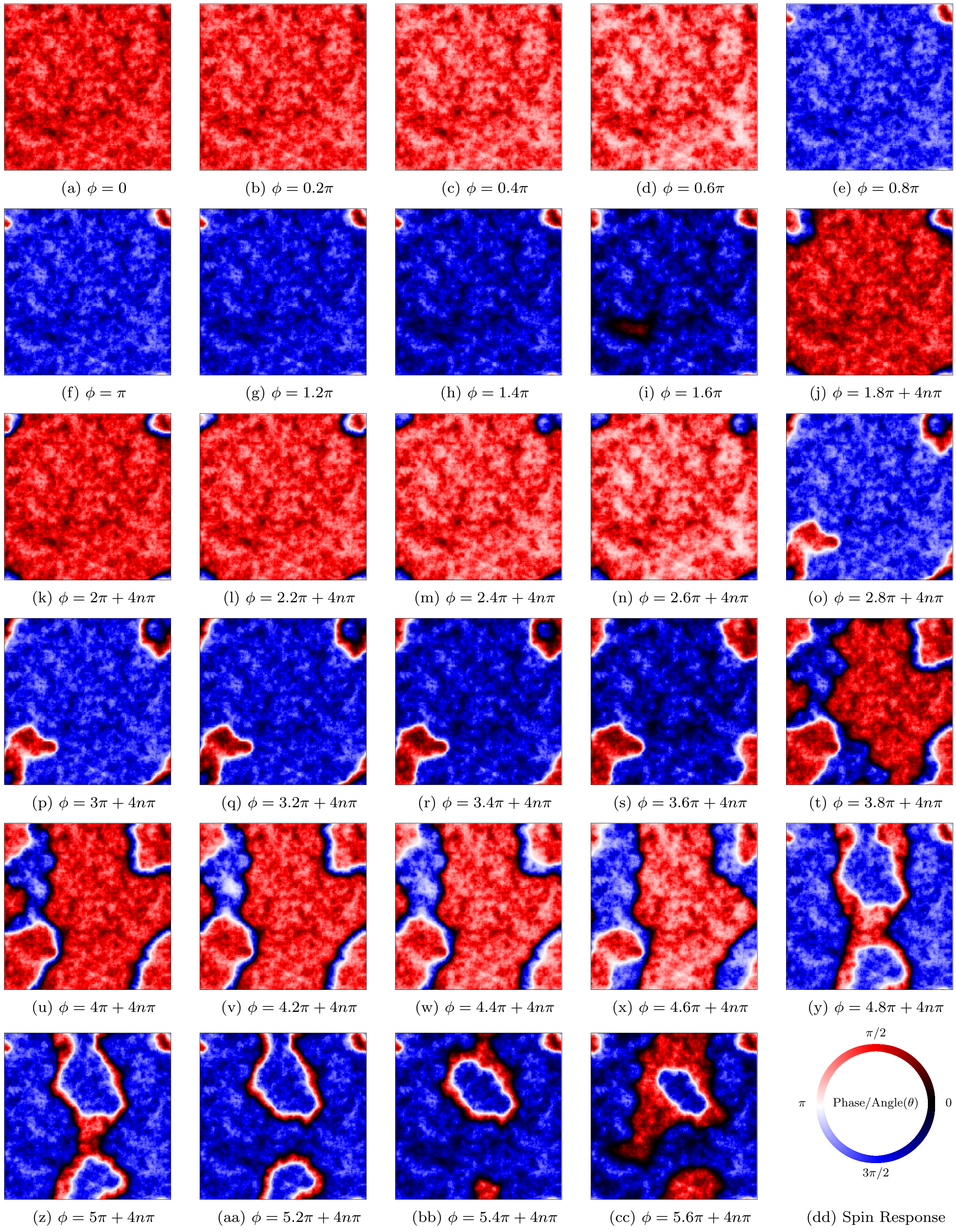

(a) $\phi = 0$ (b) $\phi = 0.2\pi$ (c) $\phi = 0.4\pi$ (d) $\phi = 0.6\pi$ (e) $\phi = 0.8\pi$

(f) $\phi = \pi$ (g) $\phi = 1.2\pi$ (h) $\phi = 1.4\pi$ (i) $\phi = 1.6\pi$ (j) $\phi = 1.8\pi + 4n\pi$

(k) $\phi = 2\pi + 4n\pi$ (l) $\phi = 2.2\pi + 4n\pi$ (m) $\phi = 2.4\pi + 4n\pi$ (n) $\phi = 2.6\pi + 4n\pi$ (o) $\phi = 2.8\pi + 4n\pi$

(p) $\phi = 3\pi + 4n\pi$ (q) $\phi = 3.2\pi + 4n\pi$ (r) $\phi = 3.4\pi + 4n\pi$ (s) $\phi = 3.6\pi + 4n\pi$ (t) $\phi = 3.8\pi + 4n\pi$

(u) $\phi = 4\pi + 4n\pi$ (v) $\phi = 4.2\pi + 4n\pi$ (w) $\phi = 4.4\pi + 4n\pi$ (x) $\phi = 4.6\pi + 4n\pi$ (y) $\phi = 4.8\pi + 4n\pi$

(z) $\phi = 5\pi + 4n\pi$ (aa) $\phi = 5.2\pi + 4n\pi$ (bb) $\phi = 5.4\pi + 4n\pi$ (cc) $\phi = 5.6\pi + 4n\pi$ (dd) Spin Response

FIG. 1. Example of spin configurations during a period-2 limit cycle with transient response of less than 2π. Spin configurations (a-i) is transient response which does repeat. Spin configurations (j-cc) are for limit cycle of 4π period which is double the periodicity of the driving field angle ϕ.

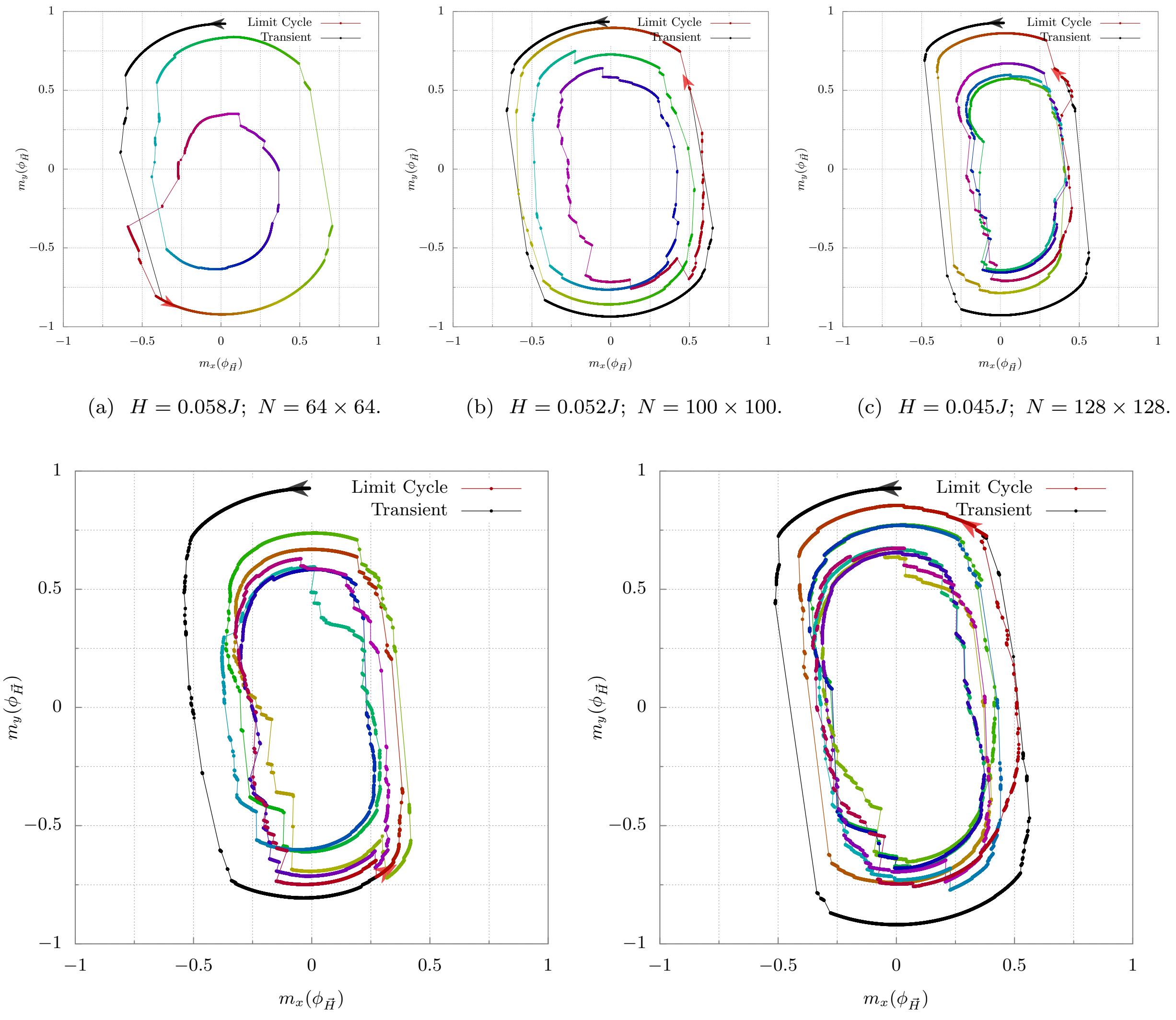

(a) $H = 0.058J$; $N = 64 \times 64$. (b) $H = 0.052J$; $N = 100 \times 100$. (c) $H = 0.045J$; $N = 128 \times 128$.

(d) $H = 0.044J$; $N = 160 \times 160$. (e) $H = 0.048J$; $N = 160 \times 160$.

FIG. 2. Transient response and multiperiod limit cycles near the transition with finite temperature fluctuation. These are results from simulations with the protcol decribed in Sec. B at T=0.1J for the Monte Carlo sweeps in between spin relaxation steps. Panels (a-e) show the initial transient response (black curves), followed by multiperiodic limit cycles (rainbow curves). These cycles are repeated for several periods and all of them stay in the same limit cycle as its zero-temperature counterpart till the end of the simulations. (a) Here, the period-2 limit cycle continues for $\Delta\phi = 375 * 2\pi$ (b) Here, the period-3 limit cycle continues for $\Delta\phi = 300 * 2\pi$ (c) Here, the period-4 limit cycle continues for $\Delta\phi = 250 * 2\pi$ (d) Here, the period-5 limit cycle continues for $\Delta\phi = 221 * 2\pi$ (e) Here, the period-7 limit cycle continues for $\Delta\phi = 230 * 2\pi$

we need to simulate in order to have at least a 1% chance that an event as rare as $|h_i| > 5R_x$ happens? The answer is a system of size at least $N \gtrsim 132 \times 132$.

This can be seen as follows. The probability that there is at least one site $i \in N$ for which $|h_i| > aR$

$$P(\exists i \in N \text{ s.t. } |h_i| > aR) \tag{2}$$

is equal to the complement of the probability that $|h_i| \leq aR, \ \forall i$:

$$P(\exists i \in N \text{ s.t. } |h_i| > aR) = 1 - P(|h_i| \leq aR, \ \forall i), \tag{3}$$

and

$$\begin{aligned} P(|h_i| \leq aR, \ \forall i) &= [P(|h_i| \leq aR)]^N \\ &= [\mathrm{erf}(a/\sqrt{2})]^N \end{aligned} \tag{4}$$

where

$$\mathrm{erf}(x) = \int_{-x}^{x} \frac{1}{\sqrt{\pi}} \exp(-y^2) dy \tag{5}$$

is the error function.

Then the required system size to have a 1% chance for such an event to occur is given by:

$$N = L^d = \frac{\log(1 - [P(\exists i \in N \text{s.t.} |h_i| > aR) \equiv 0.01])}{\log(\text{erf}(a/\sqrt{2}))} \quad (6)$$

With $a = 5$ and $d = 2$, we find that $L \geq 132$.

D. Equilibrium Results

In this section, we report our results from Monte Carlo simulations of Equation (1) in the main text in thermal equilibrium. We employ a Metropolis algorithm with checkerboard updates, in which one Monte-Carlo sweep (MCS) updates black sites and then white sites. We follow a field-cooling protocol in which the system is started at high temperature of $T = 2J$, then we reduce the temperature in steps of $\Delta T = 0.05J$ until $T = 0.05J$. At each temperature step, we thermalize the system with 128,000 MCS and then take 12,800 measurements which are taken randomly between 1 MCS and 16 MCS.

It is known that the presence of uniaxial random field disorder in the x direction ($R_x > 0$) favors spontaneous symmetry breaking in the form of ferromagnetic order in the y direction,[4–7] via an order-by-disorder mechanism. Bera *et al.* have used mean-field theory on the classical XY magnet to argue that the order-by-disorder phenomenon is robust against applied uniform magnetic field. [8] Indeed, our simulations at moderate uniaxial random field strength $R_x = 0.5J$ are consistent with spontaneous symmetry breaking in the y direction, and indicate that this phase is rather robust against disorder strength. In Fig. 3, we show that the magnetic susceptibility in the y direction diverges with system size at the transition temperature $T_c = 0.96J$ determined from the Binder parameter.

This order-by-disorder transition is robust even against uniform field applied parallel to the uniaxial random field. Our simulations of cooling in uniform applied field parallel the uniaxial random field direction (see Fig. 4) show that an order parameter develops in the direction perpendicular to the uniaxial random field, even in the presence of an applied field. This shows that the spontaneous magnetization m_y is robust even for moderate random field $R_x = 0.5J$, and finite uniform applied field H_x, as shown in Fig. 4(b), consistent with the mean field results of Ref. [8].

With strong enough transverse applied field H_x, the order-by-disorder phenomenon must be suppressed and the system will remain in the paramagnetic phase. Fig. 5 shows this crossover of the dominant magnetization from the y-axis to the x-axis with increasing applied transverse field.

[1] S. Basak, E. W. Carlson, and K. Dahmen, (2019), https://doi.org/10.4231/B90H-VH37.

[2] K. Dahmen and J. P. Sethna, Physical Review B **53**, 14872 (1996).

[3] R. A. White, Y. Liu, and K. A. Dahmen, EPL (Europhysics Letters) **86**, 50001 (2009).

[4] B. J. Minchau and R. A. Pelcovits, Phys. Rev. B **32**, 3081 (1985).

[5] D. É. Fel'dman, Journal of Experimental and Theoretical Physics **88**, 1170 (1999).

[6] J. Wehr, A. Niederberger, L. Sanchez-Palencia, and M. Lewenstein, Phys. Rev. B **74**, 224448 (2006).

[7] N. Crawford, EPL (Europhysics Letters) **102**, 36003 (2013).

[8] A. Bera, D. Rakshit, M. Lewenstein, A. Sen(De), U. Sen, and J. Wehr, Phys. Rev. B **90**, 174408 (2014).

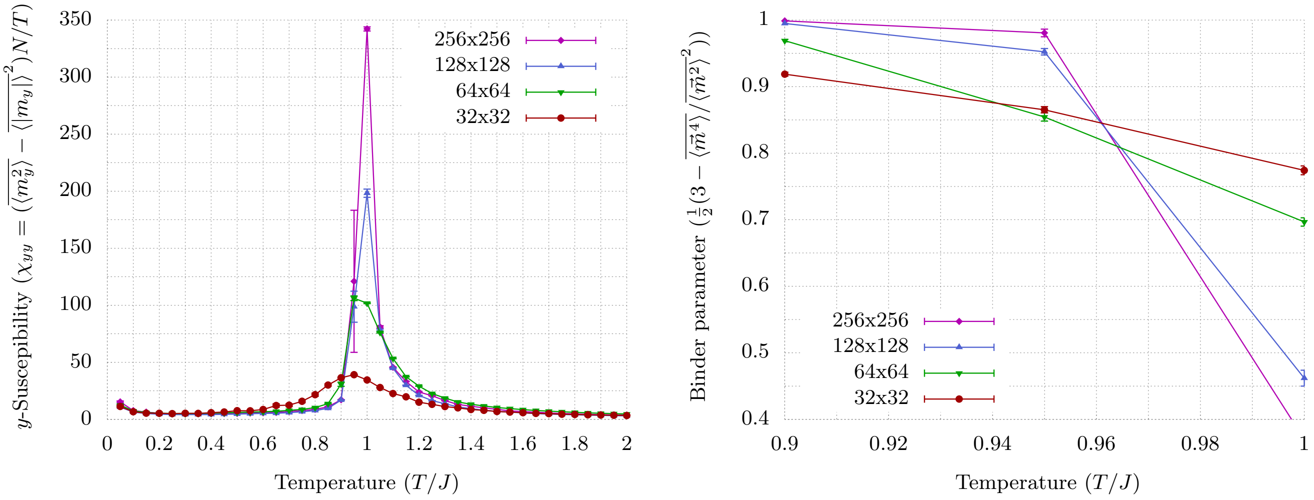

(a) Susceptibility of y-magnetization at $R_x = 0.5J$ (b) Binder parameter *vs.* temperature, at $R_x = 0.5J$

FIG. 3. Susceptibility to order and Binder parameter at moderate uniaxial disorder strength, $R_x = 0.5J$. (a) The magnetic susceptibility (χ_{yy}) in the y direction peaks near $T_c \simeq J$, and diverges as system size is increased. (b) The Binder parameter yields a transition temperature $T_c \simeq 0.96J$, consistent with the peak in the magnetic susceptibility shown in panel (a).

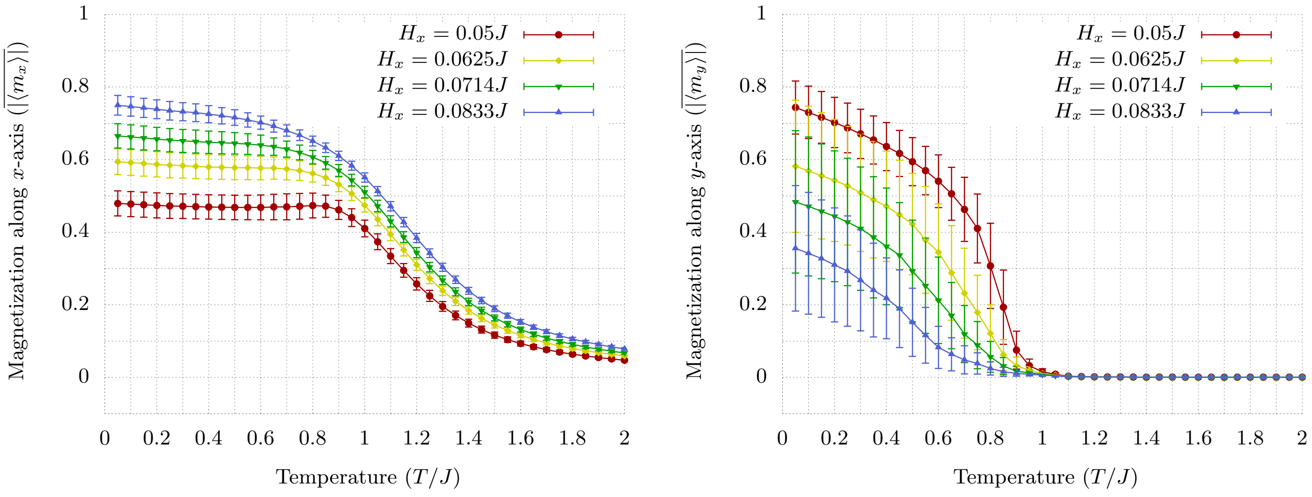

(a) $R_x = 0.5, R_y = 0.0, m_x(T), \ N = 128^2$ (b) $R_x = 0.5, R_y = 0.0, m_y(T), \ N = 128^2$

FIG. 4. Transverse field cooling at $R_x = 0.5J$. (a) Magnetization in the x direction m_x and (b) magnetization in the y direction m_y in the presence of both uniaxial random field disorder R_x *and* an applied uniform field H_x. The spontaneous magnetization m_y remains robust at finite disorder strength and in the presence of a uniform field applied transverse to the ordering direction.

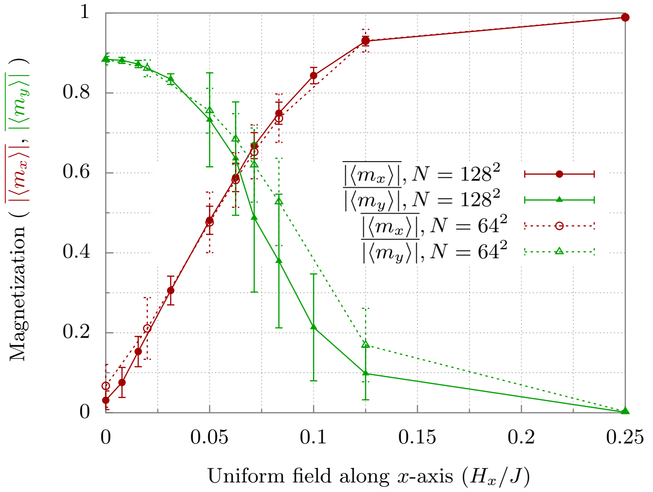

(a) $T = 0.05J,\ R_x = 0.5J,\ R_y = 0$

FIG. 5. Field cooling. Equilibrium, field-cooled magnetizations in the x and y direction, with applied field along the axis of the random field disorder $\vec{H} \parallel R_x$ with $R_x = 0.5J$, as described in the text. The horizontal axis is the value of the applied uniform field H_x during the field-cooling protocol. Upon field cooling with $H_x \lesssim R_x/10$, the net magnetization in the y direction m_y dominates over the net magnetization in the x direction m_x. This illustrates the robustness of the spontaneous magnetization in the y direction even in the presence of an applied transverse field.
