# Supplementary material for: Period multiplication cascade at the order-by-disorder transition in uniaxial random field XY magnets
Source: Nat Commun. 2020 Sep 16;11:4665. doi: 10.1038/s41467-020-18270-6 (PMC7495492; doi:10.1038/s41467-020-18270-6)
Supplement: Supplementary file 3 — Description of Additional Supplementary Files [file 41467_2020_18270_MOESM3_ESM.pdf]

Title: Supplementary Movie 1

Description: Spin configurations of the uniaxial random field XY model in the presence of a rotating driving field. In multiperiod limit cycles, it takes multiple sweeps of the drive before the spin configuration repeats. Note that in these cases, the domain walls are pinned at different locations for different cycle numbers of the drive.
